# Fatal Outcomes of COVID-19 in Patients with Severe Acute Kidney Injury

**DOI:** 10.3390/jcm9061718

**Published:** 2020-06-03

**Authors:** Jeong-Hoon Lim, Sun-Hee Park, Yena Jeon, Jang-Hee Cho, Hee-Yeon Jung, Ji-Young Choi, Chan-Duck Kim, Yong-Hoon Lee, Hyewon Seo, Jaehee Lee, Ki Tae Kwon, Shin-Woo Kim, Hyun-Ha Chang, Yong-Lim Kim

**Affiliations:** 1 Division of Nephrology, Department of Internal Medicine, School of Medicine, Kyungpook National University, Daegu 41944, Korea; jh-lim@knu.ac.kr (J.-H.L.); sh-park@knu.ac.kr (S.-H.P.); jh-cho@knu.ac.kr (J.-H.C.); hy-jung@knu.ac.kr (H.-Y.J.); jyss1002@hanmail.net (J.-Y.C.); drcdkim@knu.ac.kr (C.-D.K.); 2 Department of Statistics, Kyungpook National University, Daegu 41566, Korea; yeahnah@naver.com; 3 Division of Pulmonology and Critical Care Medicine, Department of Internal Medicine, School of Medicine, Kyungpook National University, Daegu 41944, Korea; id0121@naver.com (Y.-H.L.); dallyu17@hanmail.net (H.S.); jaelee@knu.ac.kr (J.L.); 4 Division of Infectious Disease, Department of Internal Medicine, School of Medicine, Kyungpook National University, Daegu 41944, Korea; idktkwon@gmail.com (K.T.K.); ksw2kms@knu.ac.kr (S.-W.K.)

**Keywords:** acute kidney injury, AKI severity, COVID-19, mortality, renal replacement therapy

## Abstract

The outcome of coronavirus disease 2019 (COVID-19) is associated with organ damage; however, the information about the relationship between acute kidney injury (AKI) and COVID-19 is still rare. We evaluated the clinical features and prognosis of COVID-19 patients with AKI according to the AKI severity. Medical data of hospitalized COVID-19 patients in two university-based hospitals during an outbreak in Daegu, South Korea, were retrospectively analyzed. AKI and its severity were defined according to the Acute Kidney Injury Network. Of the 164 hospitalized patients with COVID-19, 30 patients (18.3%) had AKI; 14, 4, and 12 patients had stage 1, 2, and 3, respectively. The median age was significantly higher in AKI patients than in non-AKI patients (75.5 vs. 67.0 years, *p* = 0.005). There were 17 deaths (56.7%) among AKI patients; 4 (28.6%), 1 (25.0%), and 12 (100.0%), respectively. In-hospital mortality was higher in AKI patients than in non-AKI patients (56.7% vs. 20.8%, *p* < 0.001). After adjusting for potential confounding factors, stage 3 AKI was associated with higher mortality than either non-AKI or stage 1 AKI (hazard ratio (HR) = 3.62 (95% confidence interval (CI) = 1.75–7.48), *p* = 0.001; HR = 15.65 (95% CI = 2.43–100.64), *p* = 0.004). Among the AKI patients, acute respiratory distress syndrome and low serum albumin on admission were considered independent risk factors for stage 3 AKI (both *p <* 0.05). Five patients with stage 3 AKI underwent dialysis and eventually died. In conclusion, COVID-19 patients with severe AKI had fatal outcomes.

## 1. Introduction

The new coronavirus, named severe acute respiratory syndrome coronavirus 2 (SARS-CoV-2), which originated in Wuhan, China, in December 2019 is spreading rapidly worldwide [1,2,3]. The World Health Organization declared the coronavirus disease 2019 (COVID-19) pandemic on 11 March 2020 and countries around the world are trying to prevent the spread of this virus [4]. However, the spread of infection is continuing and there is a lack of information on how to cope with it.

In February 2020, the new coronavirus spread rapidly to the community through a group worship session in a religious group named “Shincheonji” in Daegu, South Korea. The new coronavirus has a lower fatality rate but a higher infection rate than severe acute respiratory syndrome coronavirus (SARS-CoV) and Middle East respiratory syndrome coronavirus (MERS-CoV), which caused previous epidemics [5]. In particular, patients with underlying diseases, such as diabetes, hypertension, and cardiovascular disease, as well as older patients, are more likely to become infected and have a higher risk of progression to a severe condition [6]. Moreover, the mortality rate is high when accompanied by organ dysfunctions, such as in the kidneys and lungs [7].

The close relationship between acute kidney injury (AKI) and coronavirus infection has previously been identified in SARS-CoV and MERS-CoV epidemics. The reported incidence of AKI in SARS-CoV was 6.7%, whereas the AKI prevalence was up to 43.0% in MERS, which had a higher fatality than SARS-CoV [8]. Patients with severe infection, comorbid disease, and organ failure were associated with AKI occurrence, and mortality was high in these AKI patients (SARS-CoV, 91.7%; MERS-CoV, 70.0%) [9,10].

Regarding COVID-19, there is limited information on accompanying AKI; therefore, we examined the clinical course and prognosis of patients according to AKI severity. This study aimed to share the clinical features and treatment experience of COVID-19 patients with AKI.

## 2. Materials and Methods

### 2.1. Patients and Data Collection

In South Korea, COVID-19 patients who are asymptomatic and with mild severity were quarantined in therapeutic living centers assigned by the government to cope with a large number of patients and the hospital-bed shortage [11]. Patients with moderate severity were hospitalized in community hospitals, whereas severe and critical patients were admitted to designated COVID-19 treatment tertiary hospitals. We classified COVID-19 patients according to a brief telephone severity scoring system into four categories: asymptomatic to mild, moderate, severe, and critical [11]. The Kyungpook National University Hospital and Kyungpook National University Chilgok Hospital located in Daegu, South Korea, were designated as professional COVID-19 treatment tertiary hospitals for the treatment of patients of severe to critical severity. All COVID-19 patients who were admitted to these two hospitals from 17 February to 22 March 2020 were retrospectively reviewed; patient data were collected until 15 May 2020. All COVID-19 patients who had AKI during inpatient treatment were registered in this study, except those with underlying chronic kidney disease (CKD, defined as an estimated glomerular filtration rate (eGFR) of <60 mL/min/1.73 m^2^). Underlying CKD was identified using previous medical records, laboratory data, and history taking. Data on patient demographics, clinical symptoms, comorbid conditions, clinical course, and laboratory data were collected from electronic medical records. The laboratory data set included complete blood counts, liver function tests, renal function tests, and inflammatory markers. eGFR was calculated using the Chronic Kidney Disease Epidemiology Collaboration (CKD-EPI) equation [12]. The study protocol was reviewed and approved by the institutional review boards of Kyungpook National University Hospital (2020-04-059) and Kyungpook National University Chilgok Hospital (2020-04-013). The requirement for informed consent was waived because this study did not infringe on the privacy or health of the patients. All patient data were anonymized and de-identified before the analyses took place.

### 2.2. Virologic Studies

The diagnosis of COVID-19 was made through nasopharyngeal and oropharyngeal swab samples using a real-time reverse transcription polymerase chain reaction (rRT-PCR) for SARS-CoV-2. The collected samples were put into a collection tube with 150 μL of viral preservation solution and RNA was extracted with an Allplex 2019-nCoV assay (Seegen, Seoul, Korea), per the manufacturer’s instructions. Three target genes for SARS-CoV-2 were amplified and tested during each rRT-PCR assay. Detailed information for the target gene detection are as follows: RNA-dependent RNA polymerase (RdRp) gene: forward primer, 5′-GTGARATGGTCATGTGTGGCGG-3′; reverse primer, 5′-CARATGTTAAASACACTATTAGC ATA-3′; and probe in the 5-FAM/3′-BHQ format, 5′-CAGGTGGAACCTCATCAGGAGATGC-3′; E gene: forward primer, 5′-ACAGGTACGTTAATAGTTAATAGCGT-3′; reverse primer, 5′-ATATTGCAGCAGTACGCAC ACA-3′; and probe in the 5-FAM/3′-BHQ format, 5′-ACACTAGCCATCCTTACTGCGCTTCG-3′; N gene: forward primer, 5′-CACATTGGCACCCGCAATC-3′; reverse primer, 5′-GAGGAACGAGAAGAGGCTTG-3′; and probe in the 5-FAM/3′-BHQ format, 5′-ACTTCCTCAAGGAACAACATTGCCA-3′.

The RNA copy numbers were calculated using a standard curve based on the cycle threshold (Ct) values of plasmid DNA. Each dilution of plasmid DNA was tested in duplicate to produce the standard curve. The SARS-CoV-2 RNA copy numbers were converted from the Ct values of rRT-PCR. The result was considered positive if the Ct values were <37 cycles; the limit of detection was 100 copies/μL.

### 2.3. Definition

We defined AKI using the definition of the Acute Kidney Injury Network (AKIN): (a) an increase in the serum creatinine level to ≥0.3 mg/dL, (b) an increase in baseline serum creatinine level to ≥150%, or (c) the initiation of dialysis without a history of CKD [13]. The baseline serum creatinine level was defined as the serum creatinine value on admission or within 6 months before admission. The AKI stage was specified using the AKIN categories as follows: stage 1 (mild)—an increase in the baseline serum creatinine to ≥0.3 mg/dL or ≥150–199%; stage 2 (moderate)—an increase in the baseline serum creatinine level to ≥200–299%; stage 3 (severe)—an increase in baseline serum creatinine level to ≥300% or the initiation of renal replacement therapy.

Septic shock was defined using the 2016 Third International Consensus Definition for Sepsis and Septic Shock [14], and acute respiratory distress syndrome (ARDS) was defined using the Berlin Definition [15].

### 2.4. Clinical Management

All patients received supportive care, including oxygen therapy. Hospitalized COVID-19 patients were treated with (1) lopinavir/ritonavir or darunavir/cobicistat and (2) with or without hydroxychloroquine. Patients with critical COVID-19 (requiring a reservoir bag-mask oxygen supply/high-flow nasal cannula oxygen supply/mechanical ventilation or shock state) were administered with additional corticosteroid and/or intravenous immunoglobulin based on the physicians’ decision. The decision to perform renal replacement therapy was made based on the opinion of the nephrologist and infectious disease specialists.

### 2.5. Statistical Analyses

Continuous variables are presented as median (interquartile range (IQR)) values; categorical variables are presented as numbers (percentage, %). Mann–Whitney U tests were used to assess differences in continuous variables between AKI and non-AKI patients. Kruskal–Wallis tests with the post hoc Bonferroni test were used to compare the differences in continuous variables among AKI patients. Pearson chi-square tests or Fisher’s exact tests were used for categorical variables, as appropriate. The Kaplan–Meier analysis was used to estimate survival, and the log-rank test was used to analyze statistical significance. Univariate and multivariate Cox proportional hazard regression analyses were performed to evaluate the association between AKI severity and patient death. Variables that were potentially associated with mortality were entered into the multivariate analysis, namely age, sex, and complications [7]. Logistic regression analysis was used to investigate the associated factors for stage 3 AKI; the selected variables were those previously identified as risk factors for AKI [16,17]. Multivariate logistic regression analysis was performed in a stepwise manner. Variables with an alpha level of risk factor less than 0.10 in the univariate analyses were included; age and sex were included regardless of the alpha error. SPSS Statistics for Windows, version 22 (IBM Corp., Armonk, NY, USA) was used for all statistical analyses. A value of *p* < 0.05 was considered statistically significant.

## 3. Results

### 3.1. Baseline Characteristics

During the study period, 164 COVID-19 patients were hospitalized in two university-based hospitals; 34 patients matched the AKI definition (Figure S1). Of these, 4 were excluded because they had underlying CKD, and finally, 30 patients (18.3%) were classified into the AKI group and the remaining 130 patients were classified into the non-AKI group. Among the AKI group, 14 patients (46.7%) had stage 1 AKI, 4 (13.3%) had stage 2 AKI, and 12 (40.0%) had stage 3 AKI. The baseline clinical characteristics of the patients are presented in Table 1. The median age was significantly higher in the AKI group compared with the non-AKI group (75.0 vs. 67.0 years, *p* = 0.005); the male ratio was 66.7% in the AKI group and 50.8% in the non-AKI group. In the AKI group, most patients (28/30, 93.3%) had at least one comorbid disease, and the rate was significantly higher than those without AKI (93.3% vs. 73.8%, *p* = 0.02), and especially, comorbid diabetes was more common in AKI patients (46.7% vs. 27.7%, *p* = 0.04). There were 17 in-hospital deaths in the AKI group (56.7%) and 27 deaths in the non-AKI group (*p* < 0.001). In particular, all patients with stage 3 AKI died, and mortality was significantly higher in stage 3 patients than in stage 1 and stage 2 patients (stage 1: 100.0% vs. 28.6%, *p* < 0.001; stage 2: 100.0% vs. 25.0%, *p* = 0.007).

Among the AKI patients, patients commonly presented with fever (60.0%), chills (53.3%), fatigue (66.7%), cough (53.3%), sputum (46.7%), and dyspnea (56.7%); there was no difference in the signs and symptoms of patients in the different AKI groups. The median duration from symptom onset to admission was 6.5 days and that from symptom onset to AKI diagnosis was 10.0 days. The duration from symptom onset to diagnosis was longer in patients with stage 3 AKI than in those with stage 1 AKI (*p* = 0.007). Vital signs, such as blood pressure and body temperature, were similar among the groups.

### 3.2. Laboratory Findings on Admission among AKI Patients

The laboratory indices on admission among AKI patients are presented in Table 2. The median serum albumin level was 3.3 mg/dL; the level was lower in patients with stage 3 AKI than in those with stage 1 AKI (*p* = 0.01). In renal function categories, the median serum blood urea nitrogen, creatinine, and eGFR were 23.7 mg/dL, 1.1 mg/dL, and 60.0 mL/min/1.73 m^2^, respectively; they were not different between the groups.

### 3.3. Treatment and Complications among AKI Patients

The in-hospital treatments and complications among AKI patients are summarized in Table 3. About 96.7% of AKI patients were treated with lopinavir/ritonavir or darunavir/cobicistat, and 83.3% were treated with hydroxychloroquine. There was no difference in the duration of the use of these drugs among AKI groups. Stage 3 AKI patients were given inotropic agents more commonly than stage 1 AKI patients (*p* = 0.01). Stage 3 AKI patients had a higher incidence of ARDS than stage 1 AKI patients (*p* = 0.01).

### 3.4. Severity of Acute Kidney Injury and In-Hospital Mortality

**Figure 1:**
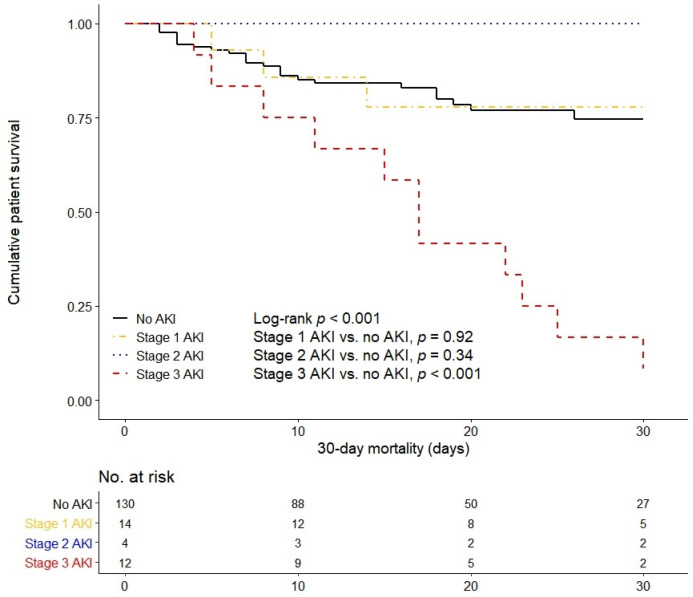
Kaplan–Meier survival curve for 30-day mortality based on the severity of acute kidney injury.

In the Kaplan–Meier survival curve, 30-day mortality was significantly higher in the stage 3 AKI group than in the non-AKI and the stage 1 and 2 AKI groups (log-rank *p <* 0.001, Figure 1). In all hospitalized COVID-19 patients, stage 3 AKI and age were independently associated with patient survival after adjusting for confounding factors, such as sex, hypertension, and diabetes (stage 3 AKI: hazard ratio (HR) = 3.62 (95% confidence interval (CI) = 1.75–7.48), *p* = 0.001; age: HR = 1.04 (95% CI = 1.01–1.07, *p* = 0.003)) (Table 4).

Among the AKI patients, the in-hospital mortality was significantly higher in stage 3 AKI patients than in stage 1 AKI patients (HR = 7.58 (95% CI = 2.04–28.24), *p* = 0.003) (Table 5). The association between stage 3 AKI and higher in-hospital mortality remained significant after adjusting for confounding factors: age and sex (model 1: HR = 7.66 (95% CI = 2.03–28.86), *p* = 0.003); age, sex, ARDS, and septic shock (model 2: HR = 15.65 (95% CI = 2.43–100.64), *p* = 0.004). In model 2, ARDS and septic shock also had an association with in-hospital mortality (ARDS: HR = 12.24 (95% CI = 1.87–79.92), *p* = 0.01; septic shock: HR = 11.42 (95% CI = 1.59–82.25), *p* = 0.02).

### 3.5. Predictors for Severe Acute Kidney Injury

In the univariate logistic regression analysis, the number of days from symptom onset to diagnosis of AKI, ARDS, septic shock, and serum albumin level on admission were associated with stage 3 AKI (all *p* < 0.05) (Table 6). In the multivariate stepwise logistic regression analysis adjusted for age and sex, ARDS and serum albumin on admission were independent predictors for stage 3 AKI (ARDS: odds ratio (OR) = 9.05 (95% CI = 1.05–78.14), *p* = 0.04; serum albumin: OR = 0.02 (95% CI = 0.001–0.87), *p* = 0.04).

### 3.6. Renal Replacement Therapy

Five out of 30 (16.7%) AKI patients underwent dialysis, all of whom were treated with continuous renal replacement therapy (CRRT) (Table 7). There were various indications for dialysis, which were as follows: hyperkalemia (2/5), hypervolemia (2/5), and hypervolemia/metabolic acidosis (1/5). The serum creatinine levels varied between 1.29 and 5.03 mg/dL. Despite dialysis, all five patients eventually died.

## 4. Discussion

This Korean COVID-19 study demonstrated that COVID-19 patients with AKI were not rare among hospitalized COVID-19 patients with severe to critical symptoms. The overall mortality of these patients was high; in particular, patients with severe AKI showed fatal outcomes and severe AKI was found to be an independent predictor of in-hospital death. Thus, clinicians should pay special attention to the treatment of COVID-19 patients with severe AKI.

Recently reported COVID-19 studies have found a prevalence rate of 0–15% for patients with AKI complicating COVID-19 [7,18,19]. The wide range of prevalence may be mainly caused by the differences in the disease severity. In this study, after excluding patients with pre-existing CKD, 18.3% of patients showed a complication with AKI among hospitalized COVID-19 patients. Based on their analyses of 116 COVID-19 patients, Wang et al. reported no AKI cases, except for those who had CKD [18]. However, patients in that study were relatively young and had mild symptoms such that the mortality in that study was only 6.0% (7/116). In contrast, the mortality of total hospitalized patients was 26.8% in our study (44/164). This difference seems to be partly associated with the characteristics of our patients. South Korea has allocated patients into professional COVID-19 treatment hospitals, community hospitals, and therapeutic living centers according to the severity score of COVID-19, which was based on age, underlying diseases (hypertension, diabetes, end-stage renal disease, congestive heart failure, chronic lung disease, and malignancy), and social factors (living in long-term care facilities and facilities for the disabled) [11]. As our hospital was designated as a professional COVID-19 treatment center, patients were screened and only those who showed a higher severity of COVID-19 were hospitalized. Therefore, patients with mild to moderate symptoms were excluded in this analysis, even if they were elderly. According to our results, the AKI incidence rate among severe or critical COVID-19 patients was up to 18%. In patients with severe to critical COVID-19, the risk of complicating AKI should be considered and frequent renal function monitoring is recommended.

Among hospitalized COVID-19 patients with severe to critical symptoms, patients who developed AKI were older and had comorbid diabetes compared with those without AKI. Previous studies have also identified these as risk factors for AKI in hospitalized and critically ill viral infection patients [20,21]. Additionally, a recent multicenter study in New York has found older age and diabetes as independent risk factors for AKI in COVID-19 patients [22]. According to our results, elderly COVID-19 patients who had diabetes need to be closely monitored for the development of AKI, and the prognosis of COVID-19 will be fatal in severe AKI patients, especially in the elderly. However, the prognosis of patients with mild to moderate AKI may not be serious.

AKI is common in hospitalized patients and is a risk factor for mortality [23]; the mortality rate is almost 70% in critically ill patients with septic AKI [24]. Stage 3 AKI patients have 6.8 times higher mortality than those without AKI among hospitalized patients [25]. Recently, Cheng et al. reported higher mortality in COVID-19 AKI patients [7]. A total of 36 out of 701 patients (5.1%) developed AKI, but among them, 14 already had CKD. After adjusting for confounding factors, stage 2 and stage 3 AKI patients were found to have 3.5 times and 4.7 times higher mortality than those with normal kidney function, respectively. However, in our study, mild and moderate AKI patients did not differ in terms of mortality; severe AKI patients had 3.6 times higher risk of in-hospital death than non-AKI patients and also had higher mortality compared with mild and moderate AKI patients. This difference might be attributable to the difference in patient characteristics and treatment. We excluded patients with CKD because the degree of AKI and the effect of AKI on prognosis could not be accurately evaluated in these patients. Moreover, many of our patients were treated with lopinavir/ritonavir or darunavir/cobicistat (29/30, 96.7%) and hydroxychloroquine (25/30, 83.3%); however, Cheng et al. reported that only 28% of the patients were given lopinavir/ritonavir without hydroxychloroquine, a potential therapeutic agent against COVID-19 [26]. A well-designed randomized controlled trial on a large patient population is required to confirm the effect of treatment on mortality.

Regarding the characteristics of AKI patients, those with severe AKI had a longer duration from symptom onset to AKI diagnosis and a worse level of inflammatory markers, such as albumin, compared with those with mild AKI. According to the results, patients with long periods from symptom onset to hospitalization, those with a longer hospitalization period, or those with severe infection signs on admission would require frequent check-ups for renal function; in particular, those with ARDS or low albumin on admission were at high risk for severe AKI.

Most AKI COVID-19 patients (28/30, 93.3%) had underlying diseases, though one severe AKI patient had no underlying disease (1/12, 8.3%; patient number 4 in Table 7). The initial serum creatinine level of this patient was normal; however, AKI occurred 1 day after admission and CRRT was initiated. Nonetheless, the disease advanced rapidly and the patient died on day 4 of hospitalization. Therefore, even if there is no underlying disease, COVID-19 patients with severe AKI have a high risk of mortality, warranting extra care from the treating doctors.

The detailed pathophysiologic relationship between COVID-19 and AKI remains unclear. However, the role of angiotensin-converting enzyme 2 (ACE2) is emerging. ACE2 has been identified as the receptor for SARS-CoV-1, and was recently confirmed as a cell entry receptor for SARS-CoV-2, potentially affecting the infectiousness of both SARS viruses [27,28]. ACE2s are expressed in many organs, especially the gastrointestinal organs and the kidney. In human tissue RNA sequencing, ACE2 expression is nearly 100 times higher in the kidneys than in the lungs [29]; thus, kidneys with abundant ACE2 expressions can be the main target of SARS-CoV-2 infection. Recent studies that have evaluated the renal histopathologic findings in COVID-19 support the direct renal infection of SARS-CoV-2 [30,31]. Additionally, a viral infection produces inflammatory mediators and can cause a cytokine storm; this results in the downstream cascade of signals and increased synthesis of proinflammatory cytokines and oxidative stress [32]. Thus, endothelial dysfunction and microvascular injury occur and cause AKI. Moreover, hypoperfusion caused by severe infection and the use of nephrotoxic agents, such as antibiotics, may contribute to AKI [9,33].

This study has certain limitations. First, the number of patients with AKI was small and the follow-up period was short; therefore, there is a possibility of type II errors. Furthermore, there was no information on the progress after discharge. Second, the Ct values of rRT-PCR could not be verified in all patients as primary screening centers often confirmed COVID-19 and transferred the confirmed patients with severe to critical symptoms to our hospitals. Third, we could not identify the effect of azithromycin that showed a reinforcing effect for hydroxychloroquine in a recent study [34]. Nonetheless, to our knowledge, this is the first study to demonstrate the risk of COVID-19 that was complicated with severe AKI in patients outside of China.

## 5. Conclusions

COVID-19 patients with severe AKI had fatal outcomes; however, the mortality rate was not increased in patients with mild and moderate AKI. Among AKI patients, those with ARDS or a low serum albumin level on admission had a higher incidence of severe AKI. Clinicians need to pay more attention to prevent and manage severe AKI in COVID-19 patients.

## Figures and Tables

**Table 1:** Clinical characteristics.

Characteristics	Non-AKI (*n* = 130)	All AKI (*n* = 30)	AKI Stage 1 (*n* = 14)	AKI Stage 2 (*n* = 4)	AKI Stage 3 (*n* = 12)
Median age (range), y	67.0 (24.0–92.0)	75.0 (60.0–98.0) *	75.0 (60.0–98.0) *	73.0 (63.0–86.0)	77.0 (61.0–87.0)
Sex, male, *n* (%)	66 (50.8)	20 (66.7)	8 (57.1)	3 (75.0)	9 (75.0)
Body mass index, kg/m^2^	23.4 (21.0–26.3)	24.5 (22.1–27.1)	25.6 (22.8–29.4)	24.0 (22.1–26.0)	23.4 (20.0–27.1)
Comorbid conditions, *n* (%)					
Any comorbidity	96 (73.8)	28 (93.3) *	14 (100.0) *	3 (75.0)	11 (91.7)
Hypertension	59 (45.4)	18 (60.0)	11 (78.6) *	2 (50.0)	5 (41.7)
Diabetes	36 (27.7)	14 (46.7) *	7 (50.0)	1 (25.0)	6 (50.0)
Cardiovascular disease	16 (12.3)	5 (16.7)	2 (14.3)	0	3 (25.0)
Congestive heart failure	8 (6.2)	2 (6.7)	0	0	2 (16.7)
Chronic lung disease	12 (9.2)	4 (13.3)	0	1 (25.0)	3 (25.0)
Cognitive disorder	26 (20.0)	4 (13.3)	3 (21.4)	0	1 (8.3)
Malignancy	19 (14.6)	7 (23.3)	2 (14.3)	1 (25.0)	4 (33.3)
Signs and symptoms, *n* (%)					
Fever	93 (71.5)	18 (60.0)	9 (64.3)	3 (75.0)	6 (50.0)
Chill	60 (46.2)	16 (53.3)	9 (64.3)	1 (25.0)	6 (50.0)
Myalgia	40 (30.,8)	9 (30.0)	5 (35.7)	0	4 (33.3)
Fatigue	63 (48.5)	20 (66.7)	10 (71.4)	2 (50.0)	8 (66.7)
Cough	45 (34.6)	16 (53.3)	7 (50.0)	1 (25.0)	8 (66.7)
Sputum	35 (26.9)	14 (46.7) *	6 (42.9)	1 (25.0)	7 (58.3) *
Dyspnea	58 (44.6)	17 (56.7)	8 (57.1)	2 (50.0)	7 (58.3)
Rhinorrhea	10 (7.7)	2 (6.7)	0	1 (25.0)	1 (8.3)
Nausea or vomiting	10 (7.7)	2 (6.7)	0	1 (25.0)	1 (8.3)
Diarrhea	19 (14.6)	3 (10.0)	2 (14.3)	1 (25.0)	0
Days from symptom onset to diagnosis	5.0 (3.0–10.0)	4.0 (3.0–7.5)	3.5 (1.0–6.3)	4.5 (1.0–6.5)	5.5 (3.5–10.5)
Days from symptom onset to admission	6.0 (3.0–10.0)	6.5 (4.0–12.0)	5.5 (3.0–10.0)	7.5 (4.5–12.0)	8.5 (5.0–15.0)
Days from symptom onset to diagnosis of AKI		10.0 (5.8–19.0)	8.0 (4.0–13.0) ^a^	8.5 (5.0–27.8) ^a,b^	17.5 (9.8–25.0) ^b^
Length of hospital stay, days	16.0 (9.0–25.0)	20.0 (12.0–31.0)	23.5 (14.0–36.0)	34.0 (10.5–57.5)	17.0 (8.8–24.5)
In-hospital death	27 (20.8)	17 (56.7) *	4 (28.6) ^a^	1 (25.0) ^b^	12 (100.0) ^*,c^
Vital signs					
Systolic BP, mmHg	133.5 (116.5–148.0)	134.0 (109.8–155.5)	115.0 (106.0–145.5)	134.5 (128.0–155.3)	150.0 (115.5–169.3)
Diastolic BP, mmHg	78.0 (68.0–86.5)	71.0 (60.0–91.3)	66.5 (59.3–84.5)	79.5 (70.5–90.0)	73.5 (59.0–98.0)
Heart rate, beats/min	88.0 (79.0–102.0)	86.0 (79.0–108.0)	79.5 (76.5–95.3)	86.5 (83.5–103.0)	91.5 (80.0–109.5)
Body temperature, °C	36.9 (36.4–37.5)	37.3 (36.8–37.9)	37.2 (36.7–38.0)	37.4 (37.0–37.7)	37.2 (36.5–38.0)
Chest radiography findings, *n* (%)					
Patchy consolidation	73 (56.2)	16 (53.3)	9 (64.3)	1 (25.0)	6 (50.0)
Ground glass opacity	64 (49.2)	15 (50.0)	6 (42.9)	2 (50.0)	7 (58.3)
No active lung lesion	24 (18.5)	4 (13.3)	2 (14.3)	1 (25.0)	1 (8.3)

* *p* < 0.05 vs. non-AKI group. The different superscripts (a, b, c) denote significant differences between groups not sharing the same superscript at the 0.05 level. Data are expressed as median (IQR) or *n* (%). Abbreviations: AKI, acute kidney injury; BP, blood pressure.

**Table 2:** Laboratory findings on admission in AKI patients.

Laboratory Findings	All AKI (*n* = 30)	AKI Stage 1 (*n* = 14)	AKI Stage 2 (*n* = 4)	AKI Stage 3 (*n* = 12)
White blood cell count, ×10^9^/L	7.1 (5.8–11.9)	7.1 (5.5–10.1)	6.0 (4.4–8.2)	9.9 (6.7–14.4)
Absolute neutrophil count, ×10^9^/L	6.3 (4.0–10.8)	5.9 (3.9–8.8)	4.9 (3.6–7.1)	9.5 (5.6–14.2)
Lymphocyte count, ×10^9^/L	0.7 (0.5–0.9)	0.9 (0.7–1.1)	0.6 (0.5–0.7)	0.6 (0.5–0.9)
Monocyte count, ×10^9^/L	0.3 (0.2–0.4)	0.3 (0.3–0.4)	0.2 (0.2–0.3)	0.3 (0.1–0.6)
Hemoglobin, g/dL	12.3 (10.9–13.9)	12.3 (10.9–13.2)	13.3 (10.8–14.7)	12.2 (10.6–13.8)
Platelet count, ×10^9^/L	241.0 (179.5–296.8)	255.5 (208.0–296.0)	204.5 (170.0–351.8)	214.0 (140.5–302.8)
hs-CRP, mg/dL	10.3 (6.1–20.9)	6.8 (4.9–16.3)	14.4 (5.8–24.4)	17.4 (8.6–26.0)
Procalcitonin, ng/mL (*n* = 16)	0.18 (0.09–0.70)	0.03 (0.02–0.19)	0.10 (0.09–0.16)	0.22 (0.15–0.91)
Ferritin, ng/mL (*n* = 19)	531.0 (255.0–868.0)	483.7 (193.3–657.6)	868.0 (484.0–920.0)	520.8 (309.0–7450.0)
AST, U/L	48.0 (36.0–63.3)	47.0 (34.5–69.3)	48.5 (38.8–50.8)	50.0 (33.0–70.5)
ALT, U/L	26.0 (17.8–34.5)	27.5 (16.3–38.3)	19.5 (19.0–24.5)	27.5 (20.3–35.5)
Total bilirubin, mg/dL	0.6 (0.4–0.9)	0.5 (0.4–0.8)	1.1 (0.6–1.4)	0.7 (0.5–0.9)
Total protein, g/dL	6.7 (6.4–7.1)	6.9 (6.6 –7.4)	6.6 (6.4–6.9)	6.5 (6.1–7.0)
Albumin, g/dL	3.3 (3.0–3.5)	3.5 (3.2–3.7) ^a^	3.5 (3.2–3.8) ^a,b^	3.2 (2.7–3.3) ^b^
BUN, mg/dL	23.7 (15.7–32.0)	23.7 (18.7–39.3)	23.3 (12.1–25.1)	23.8 (14.6–41.5)
Creatinine, mg/dL	1.1 (0.8–1.6)	1.1 (1.0–1.4)	1.1 (0.7–1.6)	1.1 (0.7–1.9)
eGFR, mL/min/1.73 m^2^	60.0 (41.5–82.0)	53.5 (41.5–69.8)	67.0 (33.8–97.3)	61.5 (38.0–86.5)
LDH, U/L (*n* = 25)	481.0 (286.0–593.0)	481.0 (249.0–589.5)	475.5 (277.0–475.5)	492.5 (306.0–592.5)
CPK, U/L (*n* = 17)	135.0 (72.5–533.5)	102.0 (63.5–436.8)	220.0 (68.0–220.0)	494.0 (177.0–837.5)
D-dimer, µg/mL (*n* = 16)	2.1 (0.8–3.2)	2.1 (1.0–2.6)	1.2 (0.2–1.2)	2.0 (0.8–6.9)
Sodium, mEq/L	135.5 (133.0–139.3)	137.0 (132.8–142.0)	135.0 (132.5–137.5)	133.5 (133.0–138.5)
Potassium, mEq/L	4.0 (3.3–4.7)	3.8 (3.5–4.9) ^a^	3.2 (3.1–3.3) ^b^	4.4 (3.6–5.2) ^a,b^
Lactate, mEq/L	1.9 (1.4–2.5)	1.9 (1.5–2.5)	1.1 (1.1–1.9)	2.0 (1.4–3.2)
Prothrombin time, s	12.4 (11.9–13.0)	12.2 (11.7–12.6)	12.6 (12.2–13.0)	12.1 (11.8–15.1)
Activated partial thromboplastin time, s	31.4 (28.7–33.5)	31.2 (26.1–34.4)	30.0 (28.6–31.2)	32.2 (30.9–34.3)

The different superscripts (a, b) denote significant differences between groups not sharing the same superscript at the 0.05 level. Data are expressed as median (IQR) or *n* (%). Abbreviations: AKI, acute kidney injury; hs-CRP, high-sensitivity C-reactive protein; AST, aspartate aminotransferase; ALT, alanine aminotransferase; BUN, blood urea nitrogen; eGFR, estimated glomerular filtration rate; LDH, lactate dehydrogenase; CPK, creatine phosphokinase.

**Table 3:** Treatment and complications in AKI patients.

Variables	All AKI (*n* = 30)	AKI Stage 1 (*n* = 14)	AKI Stage 2 (*n* = 4)	AKI Stage 3 (*n* = 12)
**Treatments, *n* (%)**				
Lopinavir/ritonavir or darunavir/cobicistat	29 (96.7)	14 (100.0)	4 (100.0)	11 (91.7)
Hydroxychloroquine	25 (83.3)	12 (85.7)	4 (100.0)	9 (75.0)
Antibiotics	29 (96.7)	13 (92.9)	4 (100.0)	12 (100.0)
Glucocorticoid	18 (60.0)	6 (42.9)	2 (50.0)	10 (83.3)
Intravenous immunoglobulin	5 (16.7)	1 (7.1)	0	4 (33.3)
Inotropic agents	17 (56.7)	5 (35.7) ^a^	2 (50.0) ^a,b^	10 (83.3) ^b^
Oxygen therapy	29 (96.7)	13 (92.9)	4 (100.0)	12 (100.0)
Low-flow oxygen	5 (16.7)	3 (21.4)	2 (50.0)	0
High-flow oxygen	8 (26.7)	5 (35.7)	0	3 (25.0)
Invasive mechanical ventilation	16 (53.3)	5 (35.7)	2 (50.0)	9 (75.0)
Continuous renal replacement therapy	5 (16.7)	0 ^a^	0 ^a,b^	5 (41.7) ^b^
Extracorporeal membrane oxygenation	1 (3.3)	0	0	1 (8.3)
ICU admission	20 (66.7)	8 (57.1)	2 (50.0)	10 (83.3)
Duration of medication used, days (IQR)				
Lopinavir/ritonavir or darunavir/cobicistat	8.0 (5.0–10.0)	7.5 (5.0–10.0)	10.5 (8.0–12.0)	8.0 (4.0–9.5)
Hydroxychloroquine	5.0 (2.0–10.0)	5.0 (2.0–10.0)	6.5 (3.0–9.5)	4.0 (1.5–9.5)
**Complications, *n* (%)**				
ARDS	17 (56.7)	5 (35.7) ^a^	2 (50.0) ^a,b^	10 (83.3) ^b^
Septic shock	14 (46.7)	4 (28.6)	1 (25.0)	9 (75.0)
Arrhythmia	2 (6.7)	1 (7.1)	0	1 (8.3)
DIC	2 (6.7)	0	0	2 (16.7)

The different superscripts (a, b) denote significant differences between groups not sharing the same superscript at the 0.05 level. Abbreviations: AKI, acute kidney injury; ARDS, acute respiratory distress syndrome; DIC, disseminated intravascular coagulation.

**Table 4:** Effect of AKI on mortality among all hospitalized COVID-19 patients.

Variables	Univariate		Model 1 ^†^		Model 2 ^‡^	
	HR (95% CI)	*p*	HR (95% CI)	*p*	HR (95% CI)	*p*
AKIN						
No AKI	Reference		Reference		Reference	
Stage 1	0.98 (0.34–2.81)	0.97	0.68 (0.23–1.98)	0.48	0.59 (0.20–1.75)	0.34
Stage 2	0.63 (0.08–4.76)	0.65	0.45 (0.06–3.42)	0.45	0.42 (0.06–3.26)	0.41
Stage 3	5.28 (2.65–10.55)	<0.001	3.77 (1.84–7.71)	<0.001	3.62 (1.75–7.48)	0.001
Age	1.04 (1.01–1.06)	0.003	1.04 (1.01–1.07)	0.003	1.04 (1.01–1.07)	0.003
Sex (ref: F)	1.52 (0.82–2.80)	0.19	0.63 (0.33–1.20)	0.16	0.61 (0.32–1.16)	0.13
Hypertension	1.44 (0.79–2.63)	0.24			1.34 (0.71–2.52)	0.36
Diabetes	1.55 (0.85–2.83)	0.15			1.35 (0.72–2.56)	0.35

^†^ Adjusted for age and sex; ^‡^ Additionally adjusted for hypertension and diabetes. Abbreviations: AKI, acute kidney injury; COVID-19, coronavirus disease 2019; HR, hazard ratio; CI, confidence interval; AKIN, Acute Kidney Injury Network.

**Table 5:** Effect of AKI on mortality among AKI patients.

Variables	Univariate		Model 1 ^†^		Model 2 ^‡^	
	HR (95% CI)	*p*	HR (95% CI)	*p*	HR (95% CI)	*p*
AKIN						
Stage 1	Reference		Reference		Reference	
Stage 2	0.46 (0.05–4.42)	0.50	0.46 (0.05–4.41)	0.50	0.07 (0.004–1.30)	0.08
Stage 3	7.58 (2.04–28.24)	0.003	7.66 (2.03–28.86)	0.003	15.65 (2.43–100.64)	0.004
Age	0.99 (0.94–1.05)	0.75	1.01 (0.94–1.07)	0.97	1.01 (0.93–1.10)	0.77
Sex (ref: F)	1.22 (0.44–3.37)	0.70	1.04 (0.33–3.26)	0.98	0.60 (0.17–2.09)	0.42
Days from symptom onset to AKI diagnosis	1.03 (0.98–1.07)	0.28				
Hypertension	0.94 (0.36–2.49)	0.94				
Diabetes	1.21 (0.45–3.22)	0.71				
ARDS	5.56 (1.57–19.75)	0.01			12.24 (1.87–79.92)	0.01
Septic shock	1.94 (0.72–5.21)	0.19			11.42 (1.59–82.25)	0.02

^†^ Adjusted for age and sex; ^‡^ Additionally adjusted for ARDS and septic shock. Abbreviations: AKI, acute kidney injury; HR, hazard ratio; CI, confidence interval; AKIN, Acute Kidney Injury Network; ARDS, acute respiratory distress syndrome.

**Table 6:** Risk factors for severe AKI among AKI patients in the logistic regression analysis.

Variables	Univariate		Multivariate ^†^	
	OR (95% CI)	*p*	OR (95% CI)	*p*
Age	0.98 (0.90–1.07)	0.65	1.01 (0.87–1.16)	0.91
Sex (ref: F)	1.91 (0.38–9.59)	0.43	1.17 (0.15–9.09)	0.88
Days from symptom onset to AKI diagnosis	1.11 (1.00–1.23)	0.04		
Hypertension	0.28 (0.06–1.29)	0.10		
Diabetes	1.25 (0.29–5.41)	0.77		
ARDS	7.86 (1.31–47.04)	0.02	9.05 (1.05–78.14)	0.04
Septic shock	7.80 (1.48–41.21)	0.02		
Serum albumin on admission	0.05 (0.004–0.69)	0.03	0.02 (0.001–0.87)	0.04

^†^ Variables with the alpha level of risk factor less than 0.10 in univariate analyses were included in the logistic regression model with backward selection; age and gender were included in the model regardless of the alpha level. Abbreviations: AKI, acute kidney injury; OR, odds ratio; CI, confidence interval; ARDS, acute respiratory distress syndrome.

**Table 7:** Information regarding patients who underwent continuous renal replacement therapy.

Patient No.	Age	Sex	SAPS	SOFA	APACHEII	Indication	Screat on CRRT Initiation (mg/dL)	Admission to CRRT Initiation (days)	Anticoagulation	Duration (hrs)	Effluent Flow Rate (mL/kg/h)	Outcome	Change to HD
1	67	M	72	12	28	Hyperkalemia	1.29	6	Heparin	155	11	Death	No
2	75	F	83	19	34	Hypervolemia	5.03	9	Heparin	560	33	Death	No
3	60	M	79	15	30	Hypervolemia and acidosis	3.47	6	Heparin	237	28	Death	No
4	67	F	57	10	19	Hypervolemia	2.82	2	Nafamostat	26	29	Death	No
5	78	M	58	10	23	Hyperkalemia	2.90	7	Nafamostat	21	25	Death	No

Abbreviations: SAPS, Simplified Acute Physiology Score; SOFA, Sequential Organ Failure Assessment; APACHE, Acute Physiology and Chronic Health Evaluation; Screat, serum creatinine; CRRT, continuous renal replacement therapy; HD, hemodialysis.

## Supplementary Materials

The following are available online at https://www.mdpi.com/2077-0383/9/6/1718/s1, Figure S1: Flow diagram of the study participants.
