# Supplementary figures and images for: Fatal Outcomes of COVID-19 in Patients with Severe Acute Kidney Injury
Source: J Clin Med. 2020 Jun 3;9(6):1718. doi: 10.3390/jcm9061718 (PMC7355571; doi:10.3390/jcm9061718)

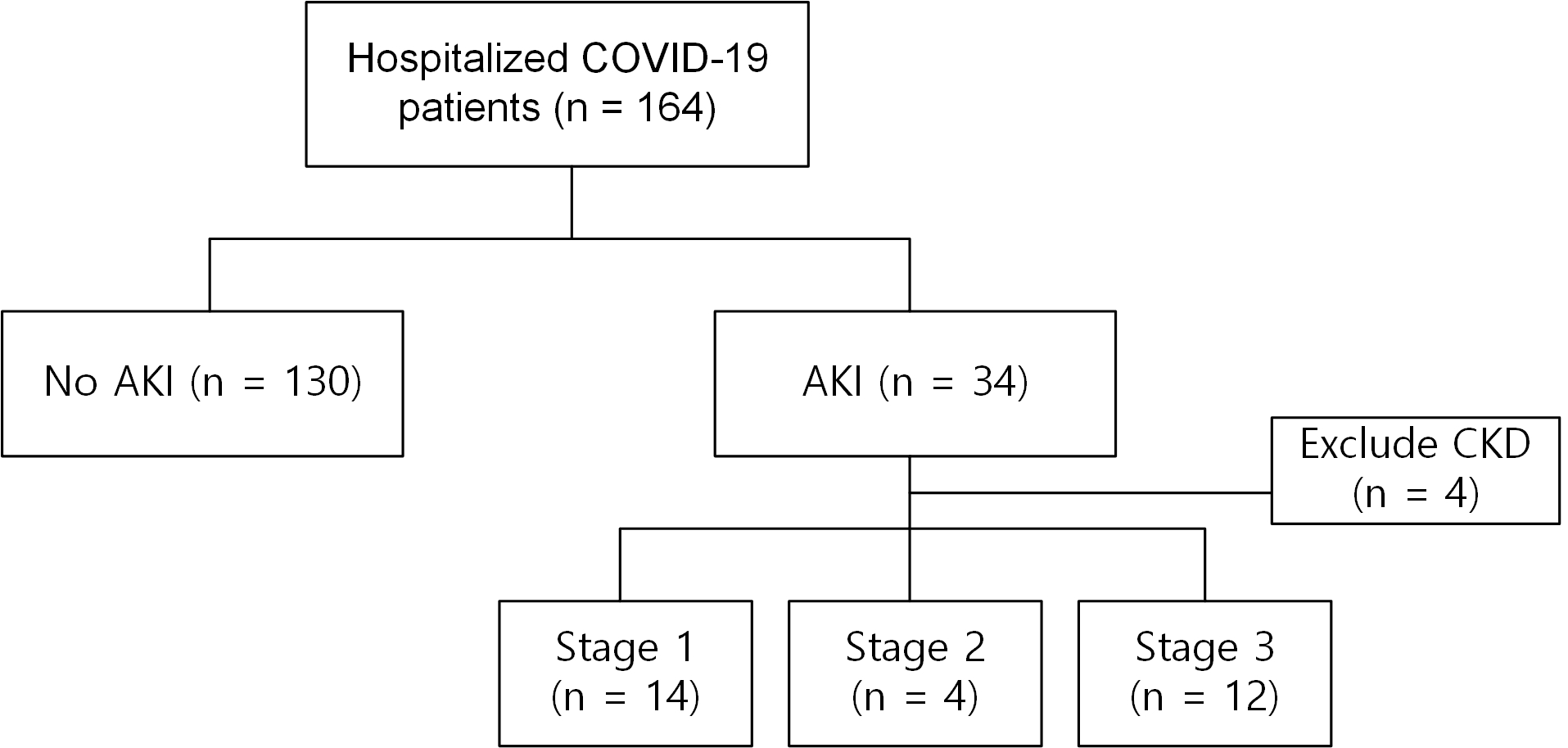

Supplement: Supplementary file 1 [file jcm-09-01718-s001.zip › jcm-806749-supplementary.jpg]
